# Supplementary material for: Antimicrobial resistance in Campylobacter jejuni and Campylobacter coli isolated from small poultry flocks in Ontario, Canada: A two-year surveillance study
Source: PLoS One. 2019 Aug 29;14(8):e0221429. doi: 10.1371/journal.pone.0221429 (PMC6715200; doi:10.1371/journal.pone.0221429)
Supplement: Attachment — Submitted filename: Response to Reviewers.docx [file pone.0221429.s001.docx]

PONE-D-19-16109

Antimicrobial resistance in Campylobacter jejuni and Campylobacter coli isolated from small poultry flocks in Ontario, Canada: A two-year surveillance study

PLOS ONE

Reviewer #1: The work presented in this manuscript is very relevant to our understanding of AMR in Campylobacter. The study is technically sound and the statistical analysis appropriate for the work. The study is particularly valuable as it was conducted before a change of regulation, which has now been implemented, and will represent a baseline for future work in the area. The authors have not overstated their conclusions and categorically indicate the limitations of the study.

Reviewer #2: The authors’ describe antimicrobial resistance (AMR) in Campylobacter from small poultry flocks in Ontario, Canada. The manuscript is well written. With a few revisions, it should be acceptable for publication.

Specific comments:

Line 49: This sentence needs a reference(s).

A reference (de Vries et al., ~11) was added as requested (Lines 49 and 360 – 363). Note that the citations and Reference List had to be updated due to the addition of this reference.

We also rewrote the sentence to make it more specific: “The transmission of antimicrobial resistant *Campylobacter* from commercial broiler and free-range chickens to humans has been described previously [11]” (Lines 48-49).

Lines 78-79: I searched for Campy-Charcoal media at Bio-Media, but could not find it. Please verify the media you used and add this to the revised manuscript.

The information on Campylobacter media was verified and updated as requested:

“The cecal material was directly plated onto Campylobacter Blood Free media (Bio-Media Unlimited Ltd., Toronto, Ontario, Canada)” (Lines 78-79).

Please find the list of products available at Bio Media, as a reference:

<https://www.bio-media.ca/Bio_Media/Bio_Media_Products.htm>

Line 82: What does a presumptive Campylobacter colony look like? Please indicate this in the revised manuscript.

The description of a presumptive *Campylobacter* colony was added as requested:

“Presumptive *Campylobacter* colonies (i.e., yellowish-gray, translucent, round, 1-2 mm diameter, smooth to slightly mucoid) were selected (Lines 80-82).

Lines 153-162: The numbers presented here are confusing and don’t add up, especially on line 157 with the game birds. Can this be rewritten to clarify the number of isolates?

We verified that the numbers are correct and rewrote the sentence for clarification:

“Of 158 submissions tested for *Campylobacter*, a total of 176 isolates were recovered: 141 isolates from chicken submissions (47 pooled samples, 3 isolates recovered from each pooled sample); 21 isolates from turkey submissions (7 pooled samples, 3 isolates recovered from each pooled sample); 6 isolates from duck submissions (2 pooled samples, 3 isolates recovered from each pooled sample); and 8 isolates from game bird (pheasant and quail) submissions (3 pooled samples in total; 3 isolates recovered from each of 2 pooled samples and 2 isolates recovered from 1 of the pooled samples).” (Lines 154-160)

Lines 230-233: Most thermophillic Campylobacter are naturally competent. However, Campylobacter restricts most foreign DNA based on methylation (https://www.ncbi.nlm.nih.gov/pubmed/28855338). I don’t agree tetO can be transferred and expressed in Campylobacter from commensals. Please think about this sentence and revise.

The reference (Stevenson et al., ~37) was replaced with the reference (Kim et al., ~37) (Line 235) and the sentence was revised as requested:

“Previous studies have shown that tetracycline resistance in *Campylobacter* is generally determined by a plasmid-encoded *tet*(O) gene [35], and that this resistance gene can be transferred horizontally between *C. jejuni* and *C. coli* isolates in the intestinal tract of food animals and humans [36, 37]”. (Lines 231-235).
